# Identification and expression analysis of splice variants of mouse enabled homologue during development and in adult tissues

**DOI:** 10.1186/1471-2199-11-45

**Published:** 2010-06-17

**Authors:** Sylvie Veniere, Davy Waterschoot, Joël Vandekerckhove, Anja Lambrechts, Christophe Ampe

**Affiliations:** 1 Department of Medical Protein Research, VIB, B-9000 Ghent, Belgium; 2 Department of Biochemistry, Faculty of Medicine and Health Sciences, Ghent University, Albert Baertsoenkaai 3, B-9000 Ghent, Belgium

## Abstract

**Background:**

The Enabled/Vasodilator stimulated phosphoprotein (Ena/VASP) gene family comprises three genes in vertebrates: *Vasp*, Enabled homologue (*Enah*) and Ena-VASP like (*Evl*). *Enah *has the most complex gene structure. It has extra alternatively included exons compared to *Vasp *and *Evl*, and possibly one alternatively excluded intron S. The aim of this mapping study was to probe the occurrence of combinations of exon usage in *Enah *thereby identifying possible vertebrate ENAH splice variants. We investigated this via an *in silico *analysis and by performing a reverse transcription-polymerase chain reaction (RT-PCR) screen on mouse samples. We further probed the expression pattern of mouse Enah splice variants during development and in a selection of mouse adult tissues and mouse cell lines.

**Results:**

*In silico *analysis of the vertebrate Ena/VASP gene family reveals that birds do not have *Vasp*, while fish have two *Evl *genes. Analysis of expressed sequence tags of vertebrate *Enah *splice variants confirms that an Enah transcript without alternative exons is ubiquitously expressed, but yields only limited information about the existence of other possible alternatively spliced Enah transcripts. Via a RT-PCR screen, we provide evidence that during mouse development and in adult mice at least eight and maximally sixteen different Enah transcripts are expressed. We also show that tissues and cell lines display specific expression profiles of these different transcripts. Exons previously associated with neuronal expression of Enah splice variants are also present in other tissues, in particular in heart.

**Conclusions:**

We propose a more uniform nomenclature for alternative exons in *Enah*. We provide an overview of distinct expression profiles of mouse Enah splice variants during mouse development, in adult mouse tissues and in a subset of mouse cell lines.

## Background

**Figure 1:**
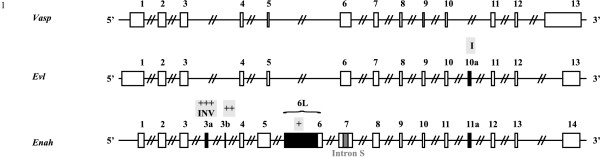
**Mouse Vasp, Evl and Enah gene structure**. *Vasp*, *Evl *and *Enah *have thirteen exons in common. The *Evl *gene has one exon in addition that can be alternatively included (exon 10a; previous name exon I). The *Enah *gene is more complex and has a longer exon 5, an extra expressed exon (*Enah *exon 6) and four alternatively included exons (exons 3a, 3b, 6L (a longer form of exon 6) and 11a; previous names +++ or INV, ++, + and 11a, respectively) and one alternatively excluded intron (intron S). An exon numbering is proposed for the alternatively included exons. The previously used exon names are also indicated.

The vertebrate Enabled/Vasodilator stimulated phosphoprotein (Ena/VASP) gene family encodes three proteins: enabled homologue (ENAH, throughout the manuscript we use protein, gene and mRNA symbols based on the format of mouse and rat nomenclature, i.e. ENAH, *Enah *and Enah, respectively) (also referred to as mammalian enabled or Mena), VASP and Ena-VASP like (EVL). Ena/VASP proteins are actin binding proteins that are involved in dynamic actin remodeling important for maintaining cell shape and cell movement (for review see [1]). These proteins are composed of four conserved domains. An N-terminal Ena/VASP homology domain 1 (EVH1) interacts with different proteins and is especially important for localization of ENA/VASP proteins in the cell. The central proline-rich region binds to the actin binding protein profilin and Src homology 3 (SH3) and WW domains. The EVH2 domain comprises the globular actin (G-actin) and filamentous actin (F-actin) binding sites and the C-terminal coiled-coil region that mediates oligomerization of the proteins. For VASP no splice variants have been reported, whereas for EVL two splice variants have been discovered: EVL and EVL-I. The latter has an insert sequence in the EVH2 domain [2]. For mouse *Enah *three alternatively included exons were initially reported: the +++ and ++ exons, located in between exons 3 and 4, and the + exon that is actually part of an alternative exon 6 due to an upstream splice event [3,4] (Figure 1). Recently, in human *Enah*, a novel alternatively included exon was found. This exon (11a) is located in between exons 11 and 12 and is at an equivalent position of exon I of mouse *Evl *[2,5]. Next to the alternatively included exons, an alternatively excluded intron has been proposed to exist in mouse *Enah*. The Enah transcript without this intron (which is part of exon 7) is referred to in literature as Mena short (Mena S) and has no proline-rich region.

ENA/VASP proteins are expressed in various tissues. VASP is highly expressed in platelets (and is the only ENA/VASP protein present there) and is also abundantly expressed in heart, stomach, intestine, spleen, lung and blood vessels [6-8]. ENAH is abundantly expressed in the brain and heart whereas it is not detectable in platelets and spleen [3,7,8]. Both mouse VASP and ENAH colocalize in stomach, intestine, kidney and heart [7]. EVL is expressed in the brain but also in spleen, thymus and testis [9].

Neuronal tissue specific expression has been reported for the mouse Enah splice variants containing exons +++, ++ or + [3]. One transcript only contains exon + whereas a second and a third transcript contain exon + combined with either exon ++ or exon +++.  Expression of human Enah transcripts containing the 11a exon is characteristic for epithelial tumor cell lines and this form appears to be involved in proliferation [5,10]. This form is down-regulated in invasive tumor cell lines whereas Enah splice variants containing alternative exons ++ and +++ are up-regulated [11,12]. Furthermore, the splice variant containing exon +++ promotes carcinoma cell motility and invasiveness *in vivo *and *in vitro*. This exon was therefore renamed INV (for invasive) [12]. The short Enah splice variant lacking intron S is preferentially expressed in spleen and B-lymphoid cell lines [13]. Also, Evl and the alternative splice variant Evl-I are differentially expressed in tissues. EVL is the predominant form in the brain, whereas EVL-I is enriched in thymus and spleen [2].

From the above studies, it is evident that attempts have been made to document expression of Enah and Evl splice variants. These studies, however, only focused on specific splice variants with only one or maximum two alternatively excluded exons. In general, information on tissue specific expression or expression during development of Enah splice variants is still lacking.

In this study we obtained a more complete view on the spectrum of expressed Enah and Evl splice variants. Next to *in silico *analysis we validate the existence of the mouse Enah splice variants by expression profiling of these transcripts during development, in tissues and in cell lines. In addition, we propose uniform and consistent names for all exons.

## Results

### ENA/VASP phylogeny

**Figure 2:**
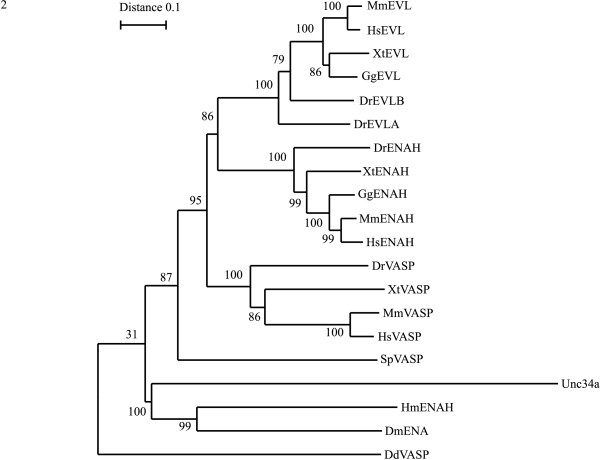
**Phylogenetic tree of Ena/VASP homologues**. The tree was constructed using the protein sequences in additional file 1: Supplemental Table S1 and was rooted on *Dictyostelium discoidum *VASP. Hs, *Homo sapiens*; Mm, *Mus musculus*; Xt, *Xenopus tropicalis*; Gg, *Gallus gallus*; Dr, *Danio rerio*; Sp, *Strongylocentrotus purpuratus*; Dd, *Dictyostelium discoidum*; Hm, *Hirudo medicinalis*; Dm, *Drosophila melanogaster*. Bootstrap values in percent are given and indicate high confidence in the vertebrate part of the three.

To investigate the general occurrence of the alternative exons described in mouse or human ENA/VASP proteins we first conducted an *in silico *analysis of ENA/VASP members. To this purpose, we systematically retrieved (predicted) protein sequences from ENSEMBL release 56 [14] and from NCBI [15] for human, mouse, chicken and the model organisms *Danio rerio *and *Xenopus tropicalis*. Because a number of these proteins were poorly or incorrectly annotated (hypothetical or by numbers) (see Additional file 1: Supplemental Table S1), we performed a phylogenetic analysis to establish their correct identity (Figure 2). For this analysis orthologues of mouse VASP, EVL and the ENAH splice variants (originally described by Gertler [3]), and selected sequences from the invertebrates *Drosophila melanogaster, Strongylocentrotus purpuratus, Hirudo medicinalis, Caenorhabditis elegans *and *Dictyostelium discoidum *were used. This search and the subsequent phylogeny yielded two surprising results. We did not find a VASP orthologue in birds, and fish have two *Evl *genes (*D. rerio *(Dr) *Evla *and *Evlb *in Figure 2) (see Additional file 1: Supplemental Results). The phylogeny also indicated that ENAH, EVL and VASP arose early in vertebrate evolution (Figure 2 and additional file 1). ENAH and EVL are more related to each other. Thus the ancestral vertebrate ENA/VASP protein appears to have first given rise to a VASP homologue and an ENAH/EVL ancestor.

### ENA/VASP gene structure

Having established the corresponding ENA/VASP homologues in the selected vertebrates we investigated conservation of their gene structures available in ENSEMBL (see Additional file 1: Supplemental Table S2). In Figure 1, we introduce a more uniform exon nomenclature. The gene structures of vertebrate *Enah*, *Vasp *and *Evl *are largely conserved, as they share thirteen coding exons with, mostly, similar start and end positions and lengths (Figure 1). Exceptions are exon 5, which is much longer in *Enah *and may encode a coiled coil domain (as predicted by SMART) and *Enah *exon 6, which is absent in *Vasp *and *Evl*. All investigated *Vasp *forms have this thirteen-exon structure. *Evl *has fourteen exons because of the presence of the extra alternatively transcribed exon (10a) between exons 10 and 11. One exception is *D. rerio Evl*, which appears to have no such exon. This extra alternatively transcribed exon is also present in vertebrate *Enah *forms (here 11a). *Enah *forms all contain seventeen exons because two additional alternatively included exons (3a and 3b) are present between exons 3 and 4. Additional transcripts can be generated by an alternative upstream splice event of exon 6, creating the longer exon 6L (exon 6L and 6 thus share the 3' part of the exon) (Figure 1). Exon 3a corresponds to the previously identified exons +++ or INV in mouse and human and exon 3b is the ++ exon in mouse. Exon 6L corresponds to the region spanning exon + and exon 6 [3-5].

Usage of the different exons adds amino acids to different parts of the ENAH protein (Additional file 2: Supplemental Figure S1). These may influence ENAH protein function in different ways. By inclusion of peptides encoded by exons 3a and 3b, nineteen and four amino acids are respectively inserted between the EVH1 domain and the proline-rich region of ENAH. This may alter the protein binding properties of the EVH1 domain and thus localization of ENAH, or change the relative position of the EVH1 domain with respect to the proline-rich domain. Insertion of exon 6L encodes an ENAH protein with a longer proline-rich region and thus a protein that may bind more profilins and/or multiple different SH3- or WW- containing proteins. ENAH with the insertion of the twenty-one amino acids encoded by exon 11a has a longer EVH2 domain. This insert is located between the actin binding motifs [3,5] and the tetramerization domain. Because of a different orientation of the actin binding region relative to the oligomerization domain, the splice variants containing these extra amino acids may have altered actin reorganization properties.

### In silico evidence for EVL and ENAH alternative splice variants

The extra exon 10a in *Evl *gives raise to two alternative splice variants. TBlastN searches using the protein sequence encoded by exon 10 and 11 with or without the EVL-I sequence encoded by exon 10a, in expressed sequence tag (EST) databases, readily revealed that both transcripts exists in human, mouse, chicken and *Xenopus tropicalis*. As mentioned above, there is no evidence from the gene structure for an EVL-I exon in *D. rerio *and EST searches also proved negative.

The *Enah *gene structure predicts that multiple splice variants of mammalian ENAH can exist. In the NCBI GenBank, however, only five mouse Enah reference sequences of transcripts are present. Transcript 4 has no alternatively included exons [GenBank: NM_001083121.1] and is usually referred to as the "general" Enah splice variant. Transcript 1 contains exons 3a and 6L [GenBank: NM_010135.2], transcript 2 has exons 3b and 6L [GenBank: NM_008680.2] and, transcript 3 exon 6L [GenBank: NM_001083120.1]. In addition, a form containing exon 11a has been deposited [Genbank: XM_001473812.1]. Similarly, in human, only two reference sequence transcripts are present: transcript 1 containing exon 11a [GenBank: NM_001008493.1], and transcript 2 is the general form [GenBank: NM_018212.4]. Chicken has one reference mRNA sequence containing exons 3b and 6L [GenBank: NM_204300.1] and *Xenopus (tropicalis and laevis) *has two reference mRNA sequences that are very similar and have no alternatively used exons [GenBank: NM_001126543.1 and NM_001092972.1]. In *D. rerio *the general form [GenBank: NM_001045028.2] is present in addition to an incomplete reference mRNA sequence [GenBank: NM_001013521.1]. Thus these reference sequences give only marginal support for the use of the alternatively included exons. We further explored their usage by searching the EST database for the presence of vertebrate Enah splice variants (see Additional file 1: Supplemental Table S3). Out of 224 mouse ESTs only one contains both exons 3a and 3b, supporting the existence of a transcript containing both of these exons. No EST with solely exon 3a was uncovered whereas we found one EST with only exon 3b. Of the other investigated organisms none had ESTs with exon 3a and/or 3b except for *X*. *tropicalis *having one EST with exon 3b. Mouse, human and chicken have, three, two and one ESTs with the exon 5_6L junction, respectively. It is of note that one of the mouse ESTs also contains exon 3b (see Additional file 1: Supplemental Table S3). There is no evidence for such ESTs in *X. tropicalis *and *D. rerio *but interestingly in these species we found three [GenBank: EL840056.1, AL961188.2, DC145972.1] and one [GenBank: EE690964.1] ESTs respectively lacking the entire exon 6. A blast search performed with exon 11a on the mouse EST database yielded five hits showing this exon is effectively used. EST searches also revealed that human (sixteen ESTs) and chicken (two ESTs) express an 11a containing form. For *Xenopus (tropicalis *and *laevis) *and *D. rerio *we did not find ESTs containing this exon. In addition, for none of the investigated organisms we found ESTs without intron S. In summary, from the EST databases there are singular observations that exon 3a is used in conjunction with exon 3b and exon 3b with exon 6L, whereas there is no evidence for combinations of 3a and 6L or 11a and 6L. Thus although the vertebrate *Enah *gene can hypothetically generate 32 alternative transcripts, little information on the potential use of the alternative exons and their combinations is present in these databases.

### Expression profiling of mouse Enah transcripts; experimental design and strategy

**Figure 3:**
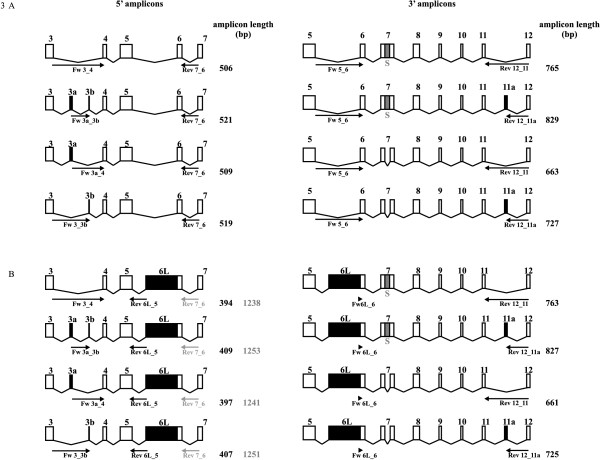
**Mouse Enah amplicons**. A. Overview of 5' and 3' amplicons with exon 6. B. Overview of 5' and 3' amplicons with exon 6L. (A-B) All theoretical amplicons are shown. Discrimination between different transcripts occurs by choice of primer pair or -when two amplicons can be formed in one RT-PCR- by amplicon length (in case of the absence or presence of intron S) via agarose gel electrophoresis.

To further map which Enah splice variants are expressed, we designed primers that are located on exon-exon boundaries at the three sites where an alternative exon can be included or excluded from the transcript (Figure 3A-B, Table 1). When such a forward and a reverse primer are combined in a RT-PCR reaction, amplification can only occur from a mRNA target that contains a certain combination of alternative exons (Figure 3A-B). Whereas this increases specificity it automatically results in primer pairs with different sensitivity. We chose, for each primer pair, to perform reactions under a certain condition (fixed total RNA concentration, fixed number of PCR cycles, see Additional file 3: Supplemental Table S4) where many samples gave a strong specific band and nonspecific amplification occurred as little as possible. However, using this approach low abundant transcripts may escape detection in a given sample. Indeed, in some samples, increasing the number of cycles reveals amplicons that were not visible under the chosen condition (Additional file 4: Supplemental Figure S2). Thus, the absence of an amplicon does not necessarily mean that the transcript is not there, but also that it may be below the detection threshold. In fact, we noticed that longer transcripts, for instance from exon-exon boundaries 3 and 4 to exon-exon boundaries 11 or 11a and 12) were difficult to amplify in a reliable manner on all samples (data not shown) whereas the same primers yielded products with primers located on exon 5 or 7 and 6/6L boundaries (see below). Therefore we divided the transcript into two groups of amplicons: 5' amplicons and 3' amplicons. The 5' amplicons potentially combine exon-exon boundaries 3_3a_3b_4 and 5_6L/6_7 and the 3' amplicons combine exon-exon boundaries 5_6L/6_7 and 11_11a_12 and eventually provide information on presence of the S intron (102 bp) via the length differences of these amplicons. Because both 5' and 3' amplicons always have either the 5_6L or the 5_6 exon-exon boundaries, we also obtain information on full length transcripts. An overview of all possible alternative exon combinations of the primers used and of the obtained amplicons is given in Figure 3A-B.

**Table 1:** Primers used for RT-PCR analysis of Enah transcripts with or without exons 3a, 3b, 6L and/or 11a.

Transcript with (+) or without (-) alternative exons:	Primer name^a^	Sequence
+ 3a and + 3b	Fw 3a_3b	5'-GCGATGTATTTTCTGTGTGTTC-3'
+ 3a and - 3b	Fw 3a_4	5'-GCGATGTATTTTCTGTGGGC-3'
- 3a and + 3b	Fw 3_3b:	5'-ATTCACAGGAAGCAGTGTTC-3'
- 3a and - 3b	Fw 3_4	5'-TTCACAGGAAGCAGGGC-3'
+ 6L	Rev 6L_5	5'-GTCTGAAGATGGAGCAGC-3'
	Rev 7_6	5'-GTCCCAAGACAAGGCCC-3'
	Fw 6L_6	5'-TTCTCCCTCTGCAGCTGC-3'
-6L	Rev 7_6	5'-GTCCCAAGACAAGGCCC-3'
	Fw 5_6	5'-AGAATGTCCAATGCTGCTG-3'
+11a	Rev 12_11a	5'-GTGTGGATTTGGGTCTGTG-3'
-11a	Rev 12_11	5'-TGTGGATTTGGGTCTGGAG-3'

^a^Format of primer name: direction (Fw = forward or Rev = reverse) followed by the numbers of the exon that form the exon boundary on which the primer is located.

### Identification of exon 6-containing Enah splice variants

**Figure 4:**
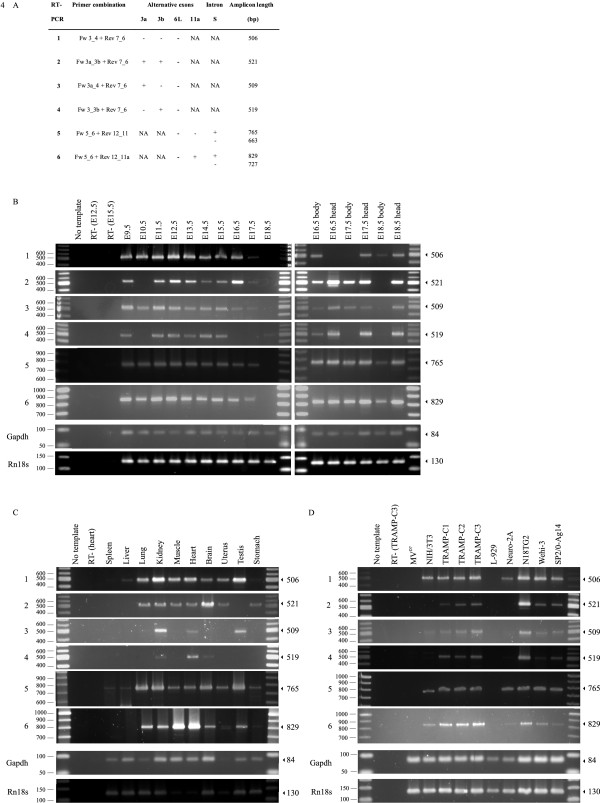
**Expression of mouse Enah transcripts with exon 6**. A. Details of the RT-PCR reactions: the primers used, the presence (+) or absence (-) of alternative exons and intron and the expected length of the amplicon in base pairs (bp) are indicated. NA, not applicable. B-C-D. The numbers 1-6 of the RT-PCR reactions on the left refer to the primer combinations in panel A. The two bottom panels are the amplifications of the household genes glyceraldehyde-3-phosphate dehydrogenase (Gapdh) and 18S ribosomal RNA (Rn18s). The molecular weight markers (in base pairs) are indicated on the left. The expected size of the amplicon in base pairs is on the right side of the panels. B. Expression of transcripts in whole mouse embryos of day E9.5 to E18.5 and in head and body fractions of E16.5 to E18.5. C. Expression of transcripts in adult mouse tissues. D. Expression of transcripts in the indicated mouse cell lines. MV^D7 ^cells are derived from the Mena/VASP double knockout mouse [27].

Theoretically, using this design sixteen different transcripts containing exon 6 (i.e. the shorter form of exon 6/6L) can be amplified (combinations of four 5' amplicons and four 3' amplicons). To maximize chances of detection, we probed expression of splice variants in a large sample set i.e. developmental stages, in various adult tissues and in cell lines of different origin. We performed four RT-PCRs (RT-PCR 1-4) to detect the four possible 5' amplicons with exon 6 and two RT-PCRs (RT-PCR 5-6) to detect the four possible 3' amplicons with exon 6 (RT-PCRs 5 and 6 each can deliver two amplicons derived from transcripts with or without intron S) (Figure 4, details in panel A). Note that in RT-PCR 1 to 4 (with primer Rev 7_6) also a product with exon 6L can be amplified (Figure 3B) but because the elongation time was optimized for the smaller amplicon, solely transcripts with exon 6 were amplified. The specificity of the primers was evident from amplification reactions on MV^D7 ^cells that do not express mouse ENAH (Figure 4D). In addition, all RT-PCRs performed in parallel (either embryonic stages, adult tissues or cell lines) included a water control (no template) and at least one sample in which the reverse transcriptase was omitted (RT-). The quality of the cDNA in each sample is evident from the amplification of glyceraldehyde-3-phosphate dehydrogenase (Gapdh) and 18S ribosomal RNA (Rn18s) (Figure 4B-C-D, two bottom panels).

We start by describing the RT-PCR-results for detection of exon 6-containing transcripts expressed in whole embryos of embryonic stages E9.5 to E18.5 and in head and body fractions of E16.5 to E18.5 (Figure 4B). All four 5' amplicons and the two 3' amplicons containing exon 6 were detected during development. We did not detect the 3' amplicons without intron S. Thus, consistent with EST presence (see above), we confirm the existence of an Enah transcript with both exons 3a and 3b (RT-PCR 2) and the existence of a transcript with exon 11a in mouse (RT-PCR 6). However, we also detected the existence of new transcripts containing either exon 3a (RT-PCR 3) or exon 3b (RT-PCR 4) together with exon 6. We observed the presence of all transcripts throughout the different stages, except at the latest stages of embryonic development in which some transcripts remain below the detection level under the conditions used (Figures 4 and additional file 4: supplemental Figure S2A, for RT-PCRs 1, 2, 4 and 6 with higher cycle number).

We performed similar RT-PCRs on different mouse adult tissues (Figure 4C). In kidney and heart we detected all four 5' amplicons and the two 3' long amplicons. In the other tissues tested (spleen, liver, lung, muscle, brain, uterus, testis and stomach) we only revealed a subset of mouse Enah transcripts. In liver and spleen, only amplicons without alternative exons are barely or not detectable: both the 5' and 3' amplicon in liver and only the 3' amplicon in spleen (RT-PCR 1 and 5). Repeating RT-PCR 1 with more amplification cycles confirms the 5' amplicon without alternative exons is also present in spleen as expected (Additional file 4: Supplemental Figure S2B). Surprisingly, although transcripts lacking intron S were shown to be preferentially expressed in spleen and also present in the brain [13], we again did not detect these transcripts in spleen, in the brain nor in any other tissue. Mouse Enah 5' variants without exon 3a and 3b (which is considered to be part of the ubiquitously expressed form), amplified in RT-PCR 1 or with both exon 3a and 3b (RT-PCR 2) are detected in most tissues, whereas 5' variants with either exon 3a (RT-PCR 3) or exon 3b (RT-PCR 4) show a more restricted and differential expression pattern. The Enah 3' amplicon without exon 11a (RT-PCR 5) (part of the ubiquitously expressed form) is detected in all tissues tested, whereas the Enah 3' amplicon with exon 11a (RT-PCR 6) is not (or hardly) detected in spleen and liver.

We also tested different mouse cell lines for expression of Enah transcripts with exon 6. The MV^D7 ^cell line derived from the Mena/VASP knockout is used as a negative control. We screened an embryonic and an adult connective tissue fibroblast cell line (NIH/3T3 and L-929, respectively), two neuroblastoma cell lines (Neuro-2a and N18TG2), the WEHI-3 leukemia cell line, the SP2/0-Ag14 myeloma cell line and three epithelial cell lines derived from a primary prostate tumor of the transgenic adenocarcinoma mouse prostate (TRAMP) model [16,17] (Figure 4D). In L-929 cells, no Enah transcripts containing exon 6 were observed (see also Additional file 4: Supplemental Figure S2B). In the NIH/3T3 and the Neuro-2a cell lines we clearly detected the 5' amplicon with no alternative exons (RT-PCR 1) and both 3' amplicons either without (RT-PCR 5) or with (RT-PCR 6) exon 11a. In addition, NIH/3T3 cells express a transcript containing exon 3a (RT-PCR 3). In the TRAMP-C, WEHI-3 and SP2/0-Ag14 cell lines all four 5' amplicons and two 3' amplicons with exon 6 are detected. Transcripts without intron S are again absent.

### Mouse Enah splice variants containing alternative exon 6L

**Figure 5:**
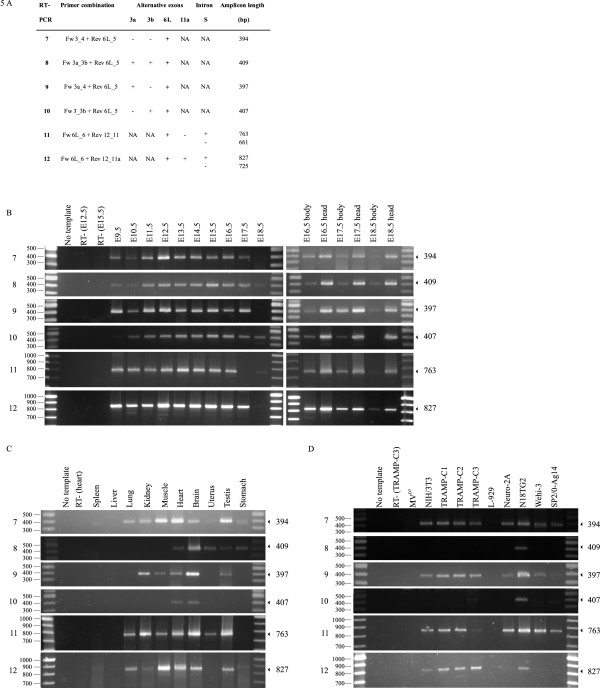
**Expression of mouse Enah transcripts with exon 6L**. A. Details of the RT-PCR reactions: the primers used, the presence (+) or absence (-) of alternative exons and intron and the expected length of the amplicon in base pairs (bp) are indicated. NA, not applicable. B-C-D. The numbers 7-12 of the RT-PCR reactions on the left refer to the primer combinations in panel A. The molecular weight markers (in base pairs) are indicated on the left. The expected size of the amplicon in base pairs is on the right side of the panels. B. Expression of transcripts in whole embryos of day E9.5 to E18.5 and in head and body fractions of E16.5 to E18.5. C. Expression of transcripts in adult mouse tissues. D. Expression of transcripts in different mouse cell lines.

Alternative exon 6L is the largest alternative exon (843 bp). This exon is translated to an extra proline-rich sequence located between the LERER region and the central proline-rich region of the ENAH protein. This exon is described to be expressed in the brain [3,18]. As is the case for transcripts with exon 6, sixteen different full length transcripts, with exon 6L potentially exist. To get insight into the presence of these transcripts we used a similar setup as in the previous section probing 5' and 3' end amplicons separately. In this case, for detection of the four possible 5' amplicons, a reverse primer overlapping exon 6L and exon 5 was used (RT-PCR 7-10), whereas for detection of the four 3' amplicons the forward primer is located in exon 6L and overlaps the 5' exon boundary of exon 6 (RT-PCR 11-12) (Figure 3B and 5A).

In samples of the developmental stages (Figure 5B) we detected six out of eight theoretical amplicons. This means that alternative exons 3a, 3b or 3a and 3b can be combined with exon 6L and that forms with exon 6L and 11a also exist. Again, we could not detect transcripts without intron S (reflected by single amplicons in RT-PCR 11 and 12).

In adult mouse tissues (Figure 5C) the 5' amplicon with solely exon 6L (RT-PCR 7) was detected in most tissues, whereas the other 5' amplicons were observed in a more limited set of tissues. This screening partially confirms the reported enrichment of amplicons with exon 6L in the brain, as six out of eight amplicons were detected in the brain: 5' amplicons with exon 6L solely (RT-PCR 7), with exon 6L and exon 3a (RT-PCR 9), with exon 6L and exon 3b (RT-PCR 10), with exon 6L, 3a and 3b (RT-PCR 8) or with exon 6L and exon 11a (RT-PCR 12). However, expression of exon 6L is not limited to the brain. This is particularly evident in heart tissue where these six amplicons with exon 6L were also found (Figure 5C). Again, in all tissues tested, transcripts without intron S were not observed. In spleen and liver no Enah transcripts with exon 6L were detected. The other tissues revealed a subset of transcripts containing exon 6L.

The mouse cell lines were also tested for the expression of splice variants containing exon 6L (Figure 5D). In L-929 cells, no Enah transcripts containing exon 6L were observed, which means that L-929 cells do not express detectable levels of Enah (Figure 4-5D). In the neuroblastoma N18TG2 cell line six out of eight amplicons containing exon 6L were detected, again amplicons without intron S were not observed. Only a subset of these six amplicons were found for the other cell lines. The 5' and 3' amplicons that contain the alternative exon 6L without exons 3a, 3b and 11a were, however, discovered in all cell lines (with the exception of L-929 cells). In addition, the splice variant containing the exons 6L and 3a was detected in these cell lines.

## Discussion

Previous expression studies have focused on the ubiquitously expressed form of mouse ENAH. Here we identify new mouse Enah transcripts that have various combinations of alternatively included exons and thus provide a more complete view of the expression profiles of distinct Enah transcripts. This is of significance for studies linking expression of certain Enah exons to proliferation or invasion of tumor cell lines [5,10-12]. Clearly, in these types of informative correlative studies combinations of used exons will have to be taken into account as well.

Protein sequences and the gene structures of vertebrate Ena/VASP homologues are relatively well conserved. Mouse *Enah *potentially gives rise to 32 different transcripts. An initial *in silico *analysis of Enah splice variants, considering all species investigated (human and the model organisms mouse, chicken, *D. rerio *and *X. tropicalis or laevis*), provided evidence for the existence of at least one EST containing either exon 3a, 3b, 6L or 11a (see Additional file 1: Supplemental Table S3). However, we found no EST evidence for the absence of the alternative intron S. It is worth noting that we could only retrieve a minimal set of ESTs containing these alternative exons. For mouse, we found 224 Enah ESTs of which only nine have alternative exons. This validates that the Enah splice variant without alternative exons is the ubiquitous form. Of these nine ESTs with alternative exons, only four different alternative exon combinations were found. One EST contains both exons 3a and 3b, one combines exons 3b and 6L, two contain only exon 6L and five have only exon 11a. Because of the limited length of EST sequences, we could not retrieve information about exon combinations 3a and/or 3b with exon 11a. Moreover, a Genbank search only retrieved five mouse Enah reference sequences, whereas in theory, if all combinations of alternative exons and introns are used, 32 Enah transcripts may exist. This indicates that the databases potentially are a poor reflection of the true complexity of the Enah transcript family. We therefore designed a RT-PCR screen to investigate the presence of mouse Enah transcripts during development, in adult tissues and in cell lines. As there might be a strong bias to the ubiquitous Enah splice variant without alternative exons (as suggested by the frequency of its occurrence in EST databases), we probed specifically for sets of transcripts using exon-specific primers. In an initial attempt we tried to amplify long Enah transcripts to get information on all possible exon combinations. We however failed to amplify these products in a reproducible manner from the various samples. Therefore we divided transcripts into two amplicons that overlap at the exons 6 or 6L. We detected, via this approach, the existence of eight Enah transcripts with different 5' region exons and four Enah transcripts with different 3' region exons (for an overview see Additional file 5: Supplemental Figure S3). This proves that at least eight and maximally sixteen mouse Enah transcripts exist. Until now, expression of variants with exon 11a was only shown in human [5]. Here, we show that mouse transcripts with exon 11a (either in combination with exon 6 or with exon 6L) are expressed in several tissues and cell lines.

Similar to the EST searches, we have no evidence for transcripts without intron S in mouse. Tani and colleagues discovered mouse Enah transcripts without intron S (in exon 7) using a similar RT-PCR set-up as the one described here [13]. They performed a RT-PCR with a forward primer located at the beginning of exon 7 and a reverse primer located in exon 8 on cDNA of mouse adult brain, spleen and on cDNA of embryonic day 17. Therefore, an amplicon representative for this splice variant should in principle be present in our RT-PCR assays 5, 6, 11 and 12. We note, however, that in spleen also other Enah transcripts are difficult to detect. It is however unclear why we did not detect this splice variant in E17.5 or in the brain.

ENAH is expressed at high levels in the developing mouse brain where it plays a role in regulation of growth cone dynamics and axon guidance [18-20]. Consistent with this, the screening of mouse embryonic stages shows that all of the identified partial transcripts were found in mouse embryonic stages and in head fractions at E18.5. In adult mice, tissue specificity has been shown for some mammalian ENAH splice variants. Mouse ENAH was detected in several tissues by immunoblotting, but given the aberrant mobility of the protein on sodium dodecyl sulphate polyacrylamide gels and the small size contributed by the coding information in exons 3a (nineteen amino acids) and 3b (four amino acids), it is unclear whether such forms can be resolved by this technique. In addition, the fact that few ESTs are present in the database suggests these transcripts are not abundantly expressed and thus likely also the protein variants will be rare. Mouse Enah splice variants with exon 6L are distinguishable from Enah splice variants with exon 6 via western blotting and are abundantly expressed in the brain [3,7,18]. We confirm that all three described variants with exon 6L are detected in the adult brain. In addition, a transcript which has exon 3a, exon 3b and exon 6L and a transcript containing both exon 6L and exon 11a are present in this tissue. However, we find that exon 6L is not exclusively used in the brain. Variants with exon 6L are also present in other tissues in particular in heart. This seems in contrast with earlier findings that ENAH with the extra sequence encoded by exon 6L was only detected via western blotting in the brain (and not in heart, kidney, any other tissue tested or in NIH/3T3 cells) [7,18,21]. RT-PCR is a more sensitive method and if appropriately designed can also detect low abundant transcripts. ENAH proteins containing the extra sequence encoded by exon 6L might be present at levels that are below detection level for western blotting. On the other hand, post-transcriptional regulation might prevent the transcripts from being translated *in vivo*.

ENAH has been shown to be abundantly expressed in heart and to be important for adult mouse heart function. Indeed, the hearts of Enah knockout mice develop structural and electrical conductivity abnormalities postnatally (unpublished data mentioned in [22]). Also, combined disruption of the localization of ENAH and VASP in cardiac intercalated disks causes dilated cardiomyopathy and early postnatal lethality, whereas VASP knock-out mice show no cardiac abnormalities [8,23,24]. Here we show that all of the discovered Enah transcripts are expressed in mouse adult heart, which suggests important specialized functions for ENAH splice variants in adult heart.

We screened mouse cell lines for Enah splice variants, and found that L-929 fibroblast cells do not detectably express Enah. NIH/3T3 fibroblast cells do, however, express several Enah transcripts, indicating differences exist within cell lines of related origin. A similar difference was shown in [5] in which six human breast tumor cell lines abundantly express ENAH, whereas in one (DAL) it is hardly detectable. All other cell lines tested express more than one Enah variant and N18TG2 cells express all eight transcripts with different 5' end and four transcripts with different 3' end and thus contain at minimum eight and possibly sixteen Enah transcripts. The three prostate cancer cells are derived from a primary prostate tumor of the transgenic adenocarcinoma mouse prostate (TRAMP) model and are of epithelial origin [16,17]. These all three express splice variants with the 11a exon which has been previously correlated with an epithelial phenotype and with proliferation of epithelial tumor cell lines [5]. Here, we in addition show that splice variants with the 11a exon are also expressed in cell lines that are not of epithelial origin. This is in line with recent findings from Warzecha and collegues [25]. Although these authors report that Enah with exon 11a is strongly present in human epithelial cells and down-regulated upon induction of epithelial-mesenchymal transition, they also show that this transcript is present at basal levels in MDA-MB-231, a cell line with a mesenchymal phenotype. Splice variants containing exon 3a (= INV) or 3b have been associated with invasion [11,12]. Forms containing this exon are present in the TRAMP C1 and C2 cell lines that are invasive *in vitro *and tumorigenic when grafted into syngeneic C57BL/6 hosts, but also in the C3 cell line that is non-tumorigenic [16,26]. Here, we show that transcripts with exons 3a, 3b or both are present in all cell lines tested (except for L-929). Expression of splice variants containing exons 3a or 3b (in combination with exon 6L) was shown in the brain indicating that expression of these exons is not limited to invasive cells [3]. We note that Enah transcripts from invasive Polyomavirus Middle T gene mouse transgenic tumors either contained the 3a or the 3b exon but a combination of these two exons was not found [11]. The TRAMP-cell lines do, however, express such forms.

## Conclusions

In summary, we report the expression of mouse Enah splice variants, caused by variable insertion of four alternative included exons (3a, 3b, 6L and 11a), in a qualitative way. By covering all possible exon combinations we provide the first strong evidence of the complexity in the ENA/VASP family on the transcript level. If we take into account all investigated developmental stages and tissues, each of these splice variants has a unique expression pattern. In different cell lines different subsets of splice variants were detected, indicating mouse tissues and cell lines have distinct profiles of Enah transcripts. We demonstrate by our approach that splice variants, identified in specific studies and attributed to specific tissues or processes, display a wider expression profile. These observations will contribute to studies into understanding ENA/VASP protein function on an organism-wide scale.

## Methods

### Cell culture

The MV^D7 ^cell line was a kind gift of Dr. F. Gertler (Massachusetts Institute of Technology) and was cultured as described [27]. NIH/3T3 embryonic fibroblast cells, SP2/0-Ag14 myeloma cells, Neuro-2a and N18TG2 neuroblastoma cells, L-929 adult connective tissue fibroblast cells and WEHI-3 leukemia cells were cultured in Dulbecco's Modified Eagle Medium (DMEM)-glutamax (Invitrogen, Carlsbad, CA) supplemented with penicillin (100 units/ml) and streptomycin, 100 μg/ml (Invitrogen, Carlsbad, CA) and heat inactivated 10% fetal bovine serum (FBS) (Hyclone-Perbio; Brackley, UK). Transgenic adenocarcinoma mouse prostate-C (TRAMP-C) cell lines (C1, C2, C3) were obtained from ATCC and cultured in DMEM containing 4 mM L-glutamine, 4.5 g/l glucose without sodium pyruvate (Invitrogen, Carlsbad, CA) supplemented with 10% FBS, 5 μg/μl bovine insulin (Sigma) and 10 nM 5 alpha-androstan-17 beta-ol-3-one (Sigma-Aldrich, St. Louis, MO).

### RNA isolation, Endpoint Reverse transcription polymerase chain reaction (RT-PCR)

Total RNA was isolated from flash-frozen mouse tissue and embryos (Swiss mice) (RNeasy Midi, Qiagen, Valencia, CA) or from collected cell lines (High Pure RNA Isolation kit) (Roche, Basel, Switzerland) according to the manufacturer's protocol. DNAse I (Qiagen or Roche) treatment was performed at room temperature for 15 min (Qiagen, Valencia, CA) or 20 min (Roche, Basel, Switzerland) depending on the kit used (RNeasy Midi, Qiagen or High Pure RNA Isolation kit, Roche) according to the manufacturer's instructions. First strand DNA synthesis was performed using 2.5 μg of RNA using the anchored -oligo(dT)_18 _primer (Transcriptor First Strand complementary DNA (cDNA) Synthesis kit) (Roche, Basel, Switzerland). Reverse transcription-polymerase chain reaction (RT-PCR) was performed in a T3000 thermocycler (Biometra, Göttingen, Germany) with Faststart taq DNA polymerase (1.5 mM MgCl_2_) (Roche, Basel, Switzerland), dNTPs (200 μM) (Invitrogen, Carlsbad, CA) and target specific primers (0.2 μM). For mouse Enah: target specific primers were designed based on [GenBank: NM_010135.2, NM_008680.2, NM_001083120.1, NM_001083121.1, BE863360.1 and CF553956.1] (Table 1) and conditions for PCR reactions with each primer set were optimized (see Additional file 4: Supplemental Table S4 for reaction conditions). Primers for household genes were designed as published [28]. The amplicons were separated on a 2% agarose gel or a 4.5% NuSieve GTG Agarose gel (Cambrex, East Rutherford, NJ) and for each amplicon a representative sample was cloned with the TOPO-TA cloning kit for sequencing (Invitrogen, Carlsbad, CA). RT-PCR product was cloned in *pCR4-TOPO *and transformed into TOP10 *E .coli *cells. Insertion of a PCR fragment causes disruption of the lethal *E. coli *gene *ccd*B and permits growth of positive recombinants. Cycle sequence reactions were performed with the BigDye Terminator v3.1 cycle sequencing kit (Applied Biosystems, Foster City, CA) in the T3000 thermocycler (Biometra, Göttingen, Germany) and analyzed with the 3100 Genetic analyzer (Applied Biosystems, Foster City, CA).

### Phylogeny

Protein sequences (see Additional file 1: Supplemental Table S1) were aligned with clustalW2 using the default settings [29,30]. The resulting alignment file was converted with Forcon [31,32] and distances were calculated with Treecon [33,34] using Poisson correction and bootstrap analysis with 500 samples. A neighbor joining tree was constructed and rooted on the *Dictyostelium discoidum *VASP sequence. Parts covering the sequences encoded by exons 3a and 3b, or 6L, or 11a flanked by the sequences encoded by the preceding and the following exon were used for TBlastN searches at NCBI in the reference gene sequence or EST sequence databases with a restriction to human, mouse, chicken (or birds), *Xenopus *(*tropicalis *and/or *laevis*) or zebrafish (or bony fish). In case of searches for 5_6L encoded sequences or sequences lacking the information of intron S the expectation value was set to 100 and low complexity filtering was not applied (see Additional file 1: Supplemental Table S1, Table S2 and Table S3 and NCBI links). The ENAH domain structure was analyzed using the SMART tool [35].

## List of abbreviations

cDNA: complementary DNA; Dd: *Dictyostelium discoidum*; Dm: *Drosophila melanogaster*; DMEM: Dulbecco's Modified Eagle Medium; Dr: *Danio rerio*; Ena: Enabled; Enah: Enabled homologue; EST: expressed sequence tag; EVH: Ena/VASP homology domain; EVL: Ena-VASP-like; F-actin: filamentous actin; FBS: fetal bovine serum; G-actin: globular actin; Gg: *Gallus gallus*; Hm: *Hirudo medicinalis*; Hs: *Homo sapiens*; INV: invasive; Mena: mammalian Ena; Mena S: Mena short; Mm: *Mus musculus*; PCR: polymerase chain reaction; RT-PCR: reverse transcription polymerase chain reaction; SH3: Src homology 3; Sp: *Strongylocentrotus purpuratus*; TRAMP-C: Transgenic adenocarcinoma mouse prostate-C; VASP: Vasodilator stimulated phosphoprotein; Xt: *Xenopus tropicalis*

## Authors' contributions

S.V. carried out the experimental work and was involved in acquisition of data, experimental design and interpretation of data. A.L. and C.A. were involved in the experimental design and interpretation of data. C.A. performed the *in silico *analysis. S.V. and C.A. wrote the manuscript. J.V. participated read and approved the final manuscript. D.W. assisted with cloning, sequencing of RT-PCR fragments and gel electrophoresis.

## Supplementary Material

Additional file 1 **Birds lack VASP and Danio rerio has two Evl genes**. supplementary results on the phylogeny of Ena/VASP proteins including: Supplemental Table S1 with the Accession numbers of protein sequences used in phylogeny, Supplemental Table S2 with Ensembl gene accession numbers of the genes of selected species and supplemental Table S3 with GenBank accession numbers of Enah EST sequences of the selected species and their source. Click here for file

Additional file 2 **Supplemental Figure S1: Hypothetical ENAH protein sequence**. ENAH protein sequence with extra sequences (encoded by alternative exon 3a, exon 3b, exon + (part of exon 6L) and exon 11a) (boxed in grey). The exon numbers are indicated above the protein sequence they encode. The sequence encoded by intron S (part of exon 7) is underlined. Amino acids sequences encoded by different exons are in black or blue (alternating). Amino acids in red are encoded by codons that lie on exon-exon boundaries. Click here for file

Additional file 3 **Supplemental Table S4: RT-PCR conditions for Enah 5' and 3'amplicons**. the table contains an overview of conditions of RT-PCR for 5' and 3'amplicons and for the household genes used in this study. Click here for file

Additional file 4 **Supplemental Figure S2: RT-PCR 1, 2, 4 and 6 with increased cycle number**. for numbering of RT-PCR reactions, see Figure 4. Data were obtained in an analogous manner as in Figure 4 except that the number of amplification cycles was 50 instead of 40 and the amount of cDNA was 100 ng. A. Expression of transcripts in whole embryos of day E9.5 to E18.5 B. Expression of transcripts in adult mouse tissues (spleen, liver and brain) and in mouse cell lines (TRAMP-C3 and L-929). The molecular weight markers (in base pairs) are indicated on the left. The expected size of the amplicon in base pairs is on the right side of the panels. Click here for file

Additional file 5 **Supplemental Figure S3: overview of Enah amplicons**. overview of RT-PCR data evidencing the presence or absence of the indicated (left) splice variant in mouse embryonic stages, adult tissues and cell lines, panel A. 5' amplicons, panel B. 3' amplicons Click here for file
