# Supplementary material for: Identification and expression analysis of splice variants of mouse enabled homologue during development and in adult tissues
Source: BMC Mol Biol. 2010 Jun 17;11:45. doi: 10.1186/1471-2199-11-45 (PMC2898656; doi:10.1186/1471-2199-11-45)
Supplement: Additional file 1 — Birds lack VASP and Danio rerio has two Evl genes. supplementary results on the phylogeny of Ena/VASP proteins including: Supplemental Table S1 with the Accession numbers of protein sequences used in phylogeny, Supplemental Table S2 with Ensembl gene accession numbers of the genes of selected species and supplemental Table S3 with GenBank accession numbers of Enah EST sequences of the selected species and their source. [file 1471-2199-11-45-S1.DOCX]

Additional File 1

*Birds lack VASP and Danio rerio has two Evl genes.*

Searches with *Mus musculus* or *Xenopus tropicalis* VASP protein sequences were negative for birds in NCBI non redundant protein (BLAST), ENSEMBL (TBLASTN) or Bird ESTs (TBLASTN) databases although each time only bird as a taxid (8782) was probed and in each case hits were obtained for ENAH or EVL sequences ([Ensembl: [ENSTGUP00000014055](http://www.ensembl.org/Taeniopygia_guttata/Transcript/ProteinSummary?db=core;g=ENSTGUG00000013651;r=Un:142273989-142274999;t=ENSTGUT00000014214)**]** annotated as VASP in Zebra Finch is actually EVL based on phylogenetic analysis). *Danio rerio* has two *Evl* genes with ample EST evidence (respectively 96 and 66 ESTs) that indicates tissue specific expression. *evla* Dr.1734: <http://www.ncbi.nlm.nih.gov/UniGene/ESTProfileViewer.cgi?uglist=Dr.17314>; *evlb* Dr.1383: <http://www.ncbi.nlm.nih.gov/UniGene/ESTProfileViewer.cgi?uglist=Dr.13833>. Two genes are also present in Fugu [Ensembl: [ENSTRUG00000010357](http://www.ensembl.org/Takifugu_rubripes/Gene/Compara_Paralog?db=core;g=ENSTRUG00000010357) and [ENSTRUG00000012665](http://www.ensembl.org/Takifugu_rubripes/Gene/Compara_Paralog?db=core;g=ENSTRUG00000012665)], in Medaka [Ensembl: [ENSORLG00000009499](http://www.ensembl.org/Oryzias_latipes/Gene/Compara_Paralog?db=core;g=ENSORLG00000009499) and [ENSORLG00000015624](http://www.ensembl.org/Oryzias_latipes/Gene/Compara_Paralog?db=core;g=ENSORLG00000015624)], Sticleback [Ensembl: [ENSGACG00000012583](http://www.ensembl.org/Gasterosteus_aculeatus/Gene/Compara_Paralog?db=core;g=ENSGACG00000012583) and [ENSGACG00000008613](http://www.ensembl.org/Gasterosteus_aculeatus/Gene/Compara_Paralog?db=core;g=ENSGACG00000008613)] and Tetraodon [Ensembl: [ENSTNIG00000010737](http://www.ensembl.org/Tetraodon_nigroviridis/Gene/Compara_Paralog?db=core;g=ENSTNIG00000010737) and [ENSTNIG00000017332](http://www.ensembl.org/Tetraodon_nigroviridis/Gene/Compara_Paralog?db=core;g=ENSTNIG00000017332)], suggesting the presence of two *Evl* genes is common among fish species.

Unfortunately no Ena/VASP member could be found in the *Ciona* database. The protein listed as VASP is actually a homer homologue. This precludes determining whether the time of duplication of *Enah/VASP* genes preceded vertebrate evolution.

Supplementary Tables

Table S1: Accession numbers of protein sequences used in phylogeny.

|  | [Database, Accession no],  annotation | | |
| --- | --- | --- | --- |
| Vertebrates | ENAH | VASP | EVL |
| Human  (*Homo sapiens*) | [GenPept: [NP_060682.2](http://www.ncbi.nlm.nih.gov/protein/39930375)]  Enabled homolog b | [Ensembl: [ENSP00000245932](http://www.ensembl.org/Homo_sapiens/Transcript/ProteinSummary?db=core;g=ENSG00000125753;r=19:50702528-50722076;t=ENST00000245932)]  VASP | [GenPept: [NP_057421](http://www.ncbi.nlm.nih.gov/protein/7706687)]^a^  ena/VASP like |
| Mouse  (*Mus musculus*) | [GenPept: [AAC52863.1](http://www.ncbi.nlm.nih.gov/protein/1644455)]^b^  mena | [Ensembl: [ENSMUSP00000032561](http://www.ensembl.org/Mus_musculus/Transcript/ProteinSummary?db=core;g=ENSMUSG00000030403;r=7:19842287-19857170;t=ENSMUST00000032561)]  VASP-201 | [Ensembl: [ENSMUSP00000105480](http://www.ensembl.org/Mus_musculus/Transcript/ProteinSummary?db=core;g=ENSMUSG00000021262;r=12:109792930-109926723;t=ENSMUST00000109854)]  EVL-203 |
| Chicken  (*Gallus gallus*) | [Ensembl: [ENSGALP37965](http://www.ensembl.org/Gallus_gallus/Transcript/ProteinSummary?db=core;g=ENSGALG00000009303;r=3:18409026-18495980;t=ENSGALT00000038757)]  IPI00819612.1 | N^c^ | [Ensembl: [ENSGALP00000018258](http://www.ensembl.org/Gallus_gallus/Transcript/ProteinSummary?db=core;g=ENSGALG00000011209;r=5:50903043-50969095;t=ENSGALT00000018280)]^d^  NP_001006487.1 |
| Xenopus  (*Xenopus tropicalis)* | [GenPept: [NP_001120015.1](http://www.ncbi.nlm.nih.gov/protein/187608763)]^e^  Enabled homolog | [Ensembl: [ENSXETP00000039549](http://www.ensembl.org/Xenopus_tropicalis/Transcript/ProteinSummary?db=core;g=ENSXETG00000018239;r=scaffold_31:3228545-3245209;t=ENSXETT00000039549)]  vasp1 | [GenPept: [NP_001096237.1](http://www.ncbi.nlm.nih.gov/protein/156717394)]  Hypothetical protein |
| Zebrafish  (*Danio rerio*) | [Ensembl: [ENSDARP00000019206](http://www.ensembl.org/Danio_rerio/Transcript/ProteinSummary?db=core;g=ENSDARG00000032049;r=20:36320614-36473790;t=ENSDART00000026539)^f^  enah] | [Ensembl: [ENSDARP00000089635](http://www.ensembl.org/Danio_rerio/Transcript/ProteinSummary?db=core;g=ENSDARG00000017105;r=18:40248688-40342736;t=ENSDART00000098865)]  vasp | [Ensembl: [ENSDARP00000051696](http://www.ensembl.org/Danio_rerio/Transcript/ProteinSummary?db=core;g=ENSDARG00000035650;r=17:28567600-28643075;t=ENSDART00000051697)],  evla  [Ensembl: [ENSDARP00000045814](http://www.ensembl.org/Danio_rerio/Transcript/ProteinSummary?db=core;g=ENSDARG00000031086;r=20:51692220-51719567;t=ENSDART00000045815)]  evlb |
| Invertebrates | Ena/VASP protein [Database, accession no], annotation | | |
| *Strongylocentrotus purpuratus* | [GenPept: [XP_781549.2](http://www.ncbi.nlm.nih.gov/entrez/query.fcgi?cmd=Retrieve&db=Protein&list_uids=115873130&dopt=GenPept&RID=HYUDDZWD013&log$=prottop&blast_rank=1)], similar to Enabled homolog | | |
| *Caenorhabditis elegans* | [GenPept: [AAN33048.1](http://www.ncbi.nlm.nih.gov/entrez/query.fcgi?cmd=Retrieve&db=Protein&list_uids=23452497&dopt=GenPept&RID=HYU2RDCP01N&log$=prottop&blast_rank=1)], Unc34a | | |
| *Dictyostelium discoidum* | [GenPept: [XP_636196.1](http://www.ncbi.nlm.nih.gov/entrez/query.fcgi?cmd=Retrieve&db=Protein&list_uids=66804927&dopt=GenPept&RID=HYU8JU8T015&log$=prottop&blast_rank=1)], vasodilator-stimulated phosphoprotein | | |
| *Hirudo medicinalis* | [GenPept: [AAG10390.1](http://www.ncbi.nlm.nih.gov/entrez/query.fcgi?cmd=Retrieve&db=Protein&list_uids=10048320&dopt=GenPept&RID=HYUA19HP013&log$=prottop&blast_rank=1)], enabled-like protein | | |
| *Drosophila melanogaster* | [GenPept: [NP_001137709.1](http://www.ncbi.nlm.nih.gov/entrez/query.fcgi?cmd=Retrieve&db=Protein&list_uids=221330427&dopt=GenPept&RID=HYUC5ZXA011&log$=prottop&blast_rank=1)], enabled isoform F | | |

^a^ This entry is EVL-I, for phylogeny the 10a encoded sequence was removed. The resulting EVL sequence was validated by several ESTs.

^b^ Reference sequence published by [3].

^c^ no protein present

^d^ This entry is EVL-I, for phylogeny the 10a encoded sequence was removed. The resulting EVL sequence was validated by more than ten ESTs.

^e^ This sequence has part of the amino acid sequence encoded by the exon 6L (and was used for phylogeny). As yet there is no EST evidence for this sequence. All ESTs of *Xenopus tropicalis* suggest that either exon 6 is skipped (three ESTs) or used (two ESTs), as well for *Xenopus laevis* (one EST with exon 6; three ESTs without exon 6).

^f^ This entry is twenty-five residues longer at the N-terminus than other ENAH orthologues, these were removed for phylogeny. Two ESTs exist for the form with these extra residues; eight EST for the form without these residues were found.

Table S2: Ensembl gene accession numbers of the selected species.

|  | *Enah* | *Vasp* | *Evl* |
| --- | --- | --- | --- |
| Human  (*Homo sapiens*) | [[ENSG00000154380](http://www.ensembl.org/Homo_sapiens/geneview?gene=ENSG00000154380)] | [[ENSG00000125753](http://www.ensembl.org/Homo_sapiens/geneview?gene=ENSG00000125753)] | [[ENSG00000196405](http://www.ensembl.org/Homo_sapiens/geneview?gene=ENSG00000196405)] |
| Mouse  (*Mus musculus*) | [[ENSMUSG00000022995](http://www.ensembl.org/Mus_musculus/geneview?gene=ENSMUSG00000022995)] | [[ENSMUSG00000030403](http://www.ensembl.org/Mus_musculus/geneview?gene=ENSMUSG00000030403)] | [[ENSMUSG00000021262](http://www.ensembl.org/Mus_musculus/geneview?gene=ENSMUSG00000021262)] |
| Chicken  (*Gallus gallus*) | [[ENSGALG00000009303](http://www.ensembl.org/Gallus_gallus/geneview?gene=ENSGALG00000009303)] | / | [[ENSGALG00000011209](http://www.ensembl.org/Gallus_gallus/geneview?gene=ENSGALG00000011209)] |
| Xenopus  (*Xenopus tropicalis)* | [[ENSXETG00000018550](http://www.ensembl.org/Xenopus_tropicalis/geneview?gene=ENSXETG00000018550)] | [[ENSXETG00000018239](http://www.ensembl.org/Xenopus_tropicalis/geneview?gene=ENSXETG00000018239)] | [[ENSXETG00000002529](http://www.ensembl.org/Xenopus_tropicalis/geneview?gene=ENSXETG00000002529)] |
| Zebrafish  (*Danio rerio*) | [[ENSDARG00000032049](http://www.ensembl.org/Danio_rerio/geneview?gene=ENSDARG00000032049)] | [[ENSDARG00000017105](http://www.ensembl.org/Danio_rerio/geneview?gene=ENSDARG00000017105)] | [[ENSDARG00000035650](http://www.ensembl.org/Danio_rerio/geneview?gene=ENSDARG00000035650)] ^a^  [[ENSDARG00000031086](http://www.ensembl.org/Danio_rerio/geneview?gene=ENSDARG00000031086)] ^b^ |

^a^ *evla*

^b^ *evlb*

Table S3: GenBank accession numbers of Enah EST sequences of the selected species and their source.

|  | Total EST number^a^ | 3a and 3b | 3a | 3b | 6L | 11a |
| --- | --- | --- | --- | --- | --- | --- |
| Human  (*Homo sapiens*) | 700 | NF^b^ | NF | NF | [[BG982642.1](http://www.ncbi.nlm.nih.gov/entrez/query.fcgi?cmd=Retrieve&db=Nucleotide&list_uids=14385377&dopt=GenBank&RID=G7FC1P8A01N&log$=nuclalign&blast_rank=1)]  colon  [[DA809712.1](http://www.ncbi.nlm.nih.gov/entrez/query.fcgi?cmd=Retrieve&db=Nucleotide&list_uids=82121365&dopt=GenBank&RID=FGPHAYV701S&log$=nuclalign&blast_rank=1)]  brain | [[DA383906.1](http://www.ncbi.nlm.nih.gov/entrez/query.fcgi?cmd=Retrieve&db=Nucleotide&list_uids=81160723&dopt=GenBank&RID=FGHKYDN101S&log$=nuclalign&blast_rank=1)]  thalamus  [[CN419174.1](http://www.ncbi.nlm.nih.gov/entrez/query.fcgi?cmd=Retrieve&db=Nucleotide&list_uids=47406768&dopt=GenBank&RID=G7HE8NP201S&log$=nuclalign&blast_rank=2)]  embryonic stem cells  [[DB125445.1](http://www.ncbi.nlm.nih.gov/entrez/query.fcgi?cmd=Retrieve&db=Nucleotide&list_uids=83231798&dopt=GenBank&RID=G7HE8NP201S&log$=nuclalign&blast_rank=3)]  thymus  [[CN419172.1](http://www.ncbi.nlm.nih.gov/entrez/query.fcgi?cmd=Retrieve&db=Nucleotide&list_uids=47406766&dopt=GenBank&RID=G7HE8NP201S&log$=nuclalign&blast_rank=4)]  embryonic stem cells  plus 12 other^c^ |
| Mouse  (*Mus musculus*) | 224 | [[BE863360.1](http://www.ncbi.nlm.nih.gov/nucest/10383262?report=genbank&log$=seqview)]  brain (mixture) | NF | [[CF744951.1](http://www.ncbi.nlm.nih.gov/entrez/query.fcgi?cmd=Retrieve&db=Nucleotide&list_uids=37641291&dopt=GenBank&RID=FFXJ9FXZ01N&log$=nuclalign&blast_rank=8)]  whole brain | [[BQ887239.1](http://www.ncbi.nlm.nih.gov/entrez/query.fcgi?cmd=Retrieve&db=Nucleotide&list_uids=22279253&dopt=GenBank&RID=FGPUHCBS01S&log$=nuclalign&blast_rank=1)]  otocysts  [[BU055676.1](http://www.ncbi.nlm.nih.gov/entrez/query.fcgi?cmd=Retrieve&db=Nucleotide&list_uids=22495753&dopt=GenBank&RID=G7FAR0EE01S&log$=nuclalign&blast_rank=2)]  whole brain  [[CF744951.1](http://www.ncbi.nlm.nih.gov/entrez/query.fcgi?cmd=Retrieve&db=Nucleotide&list_uids=37641291&dopt=GenBank&RID=G7FAR0EE01S&log$=nuclalign&blast_rank=3)]  whole brain | [[CB233777.1](http://www.ncbi.nlm.nih.gov/entrez/query.fcgi?cmd=Retrieve&db=Nucleotide&list_uids=28285355&dopt=GenBank&RID=G7GWCDGJ01S&log$=nuclalign&blast_rank=1)]  brain  [[CF553956.1](http://www.ncbi.nlm.nih.gov/entrez/query.fcgi?cmd=Retrieve&db=Nucleotide&list_uids=34890790&dopt=GenBank&RID=G7GWCDGJ01S&log$=nuclalign&blast_rank=2)]  organ of Corti ear  [[AI893955.1](http://www.ncbi.nlm.nih.gov/entrez/query.fcgi?cmd=Retrieve&db=Nucleotide&list_uids=5599857&dopt=GenBank&RID=G7GWCDGJ01S&log$=nuclalign&blast_rank=3)]  embryo  [[AV453565.1](http://www.ncbi.nlm.nih.gov/entrez/query.fcgi?cmd=Retrieve&db=Nucleotide&list_uids=140506174&dopt=GenBank&RID=G7GWCDGJ01S&log$=nuclalign&blast_rank=4)]  embryonic stem cells  [[BI653536.1](http://www.ncbi.nlm.nih.gov/entrez/query.fcgi?cmd=Retrieve&db=Nucleotide&list_uids=15567772&dopt=GenBank&RID=G7GWCDGJ01S&log$=nuclalign&blast_rank=15)]  tumor gross tissue |
| Chicken  (*Gallus gallus*) | 32 | NF | NF | NF | [[BI389815.1](http://www.ncbi.nlm.nih.gov/entrez/query.fcgi?cmd=Retrieve&db=Nucleotide&list_uids=15083097&dopt=GenBank&RID=FGPVK04U01N&log$=nuclalign&blast_rank=1)]  pituitary gland  hypothalamus  pineal gland | [[BU325974.1](http://www.ncbi.nlm.nih.gov/entrez/query.fcgi?cmd=Retrieve&db=Nucleotide&list_uids=25833975&dopt=GenBank&RID=FGM98VBW01N&log$=nucltop&blast_rank=1)]  Head  [[BU246268.1](http://www.ncbi.nlm.nih.gov/entrez/query.fcgi?cmd=Retrieve&db=Nucleotide&list_uids=25493765&dopt=GenBank&RID=G7HEERWU01S&log$=nuclalign&blast_rank=10)]  Liver |
| Xenopus  (*Xenopus tropicalis)* | 85 + 40^d^ | NF | NF | [[CX813868.1](http://www.ncbi.nlm.nih.gov/entrez/query.fcgi?cmd=Retrieve&db=Nucleotide&list_uids=58368495&dopt=GenBank&RID=FFY8771B01S&log$=nuclalign&blast_rank=1)]  brain | NF | NF |
| Zebrafish  (*Danio rerio*) | 52 | NF | NF | NF | NF | NF |

^a^ Links to all Unigene entries of Enah EST sequences of the selected species:

<http://www.ncbi.nlm.nih.gov/UniGene/clust.cgi?UGID=713052&TAXID=9606&SEARCH=enabled%20human>

<http://www.ncbi.nlm.nih.gov/UniGene/clust.cgi?ORG=Mm&CID=389224&MAXEST=224A>

<http://www.ncbi.nlm.nih.gov/UniGene/clust.cgi?UGID=2936001&TAXID=9031&SEARCH=chicken%20enabled>

<http://www.ncbi.nlm.nih.gov/UniGene/clust.cgi?UGID=462604&TAXID=8364&SEARCH=enabled%20xenopus%20tropicalis>

<http://www.ncbi.nlm.nih.gov/UniGene/clust.cgi?UGID=2873280&TAXID=8364&SEARCH=enabled%20xenopus%20tropicalis>

<http://www.ncbi.nlm.nih.gov/UniGene/clust.cgi?UGID=108725&TAXID=7955&SEARCH=enabled%20danio>

^b^ not found

^c^ none of these contains the exon 5_6L boundary

^d^ In unigene there are two different lists of ESTs referring to expression of enabled homolog [GenPept: [**NP_001120015.1**](http://www.ncbi.nlm.nih.gov/protein/187608763)**]**
