# Supplementary material for: Identification and expression analysis of splice variants of mouse enabled homologue during development and in adult tissues
Source: BMC Mol Biol. 2010 Jun 17;11:45. doi: 10.1186/1471-2199-11-45 (PMC2898656; doi:10.1186/1471-2199-11-45)
Supplement: Additional file 3 — Supplemental Table S4: RT-PCR conditions for Enah 5' and 3'amplicons. the table contains an overview of conditions of RT-PCR for 5' and 3'amplicons and for the household genes used in this study. [file 1471-2199-11-45-S3.DOCX]

Table S4: overview of conditions of RT-PCR for 5’ and 3’amplicons and household genes.

| RT-PCR number in article | Primer combination | Alternative exon combination | RT-PCR program (AT^1^; AT^2^; ET)^a^ | cDNA amount (ng) | Sequence verified? |
| --- | --- | --- | --- | --- | --- |
| 1 | Fw 3_4  Rev 7_6 |  | AT^1^=56°C; AT^2^=55°C; ET=45s | Embryo stage = 20ng  Cell line=20ng  Tissues=100ng | yes |
| 2 | Fw 3a_3b  Rev 7_6 | 3a, 3b | AT^1^=58°C; AT^2^=57°C; ET=45s | Embryo stage = 100ng  Cell line=100ng  Tissues=100ng | yes |
| 3 | Fw 3a_4  Rev 7_6 | 3a | AT^1^=58°C; AT^2^=57°C; ET=45s | Embryo stage = 100ng  Cell line=100ng  Tissues=100ng | yes |
| 4 | Fw 3_3b  Rev 7_6 | 3b | AT^1^=56°C; AT^2^=55°C; ET=45s | Embryo stage = 100ng  Cell line=100ng  Tissues=100ng | yes |
| 5 | Fw 5_6  Rev 12_11 |  | AT^1^=56°C; AT^2^=55°C; ET=60s | Embryo stage = 20ng  Cell line=20ng  Tissues=100ng | yes |
| 6 | Fw 5_6  Rev 12_11a | 11a | AT^1^=56°C; AT^2^=55°C; ET=60s | Embryo stage = 20ng  Cell line=20ng  Tissues=100ng | yes |
| 7 | Fw 3_4  Rev 6L_5 | 6L | AT^1^=56°C; AT^2^=55°C; ET=45s | Embryo stage = 20ng  Cell line=20ng  Tissues=100ng | yes |
| 8 | Fw 3a_3b  Rev 6L_5 | 3a, 3b and  6L | AT^1^=56°C; AT^2^=55°C; ET=45s | Embryo stage = 100ng  Cell line=100 ng  Tissues=100ng | yes |
| 9 | Fw 3a_4  Rev 6L_5 | 3a and  6L | AT^1^=56°C; AT^2^=55°C; ET=45s | Embryo stage = 20ng  Cell line=20ng  Tissues=100ng | yes |
| 10 | Fw 3_3b  Rev 6L_5 | 3b and  6L | AT^1^=57°C; AT^2^=56°C; ET=45s | Embryo stage = 20ng  Cell line=100 ng  Tissues=100ng | yes |
| 11 | Fw 6L_6  REV 12_11 | 6L | AT^1^=56°C; AT^2^=55°C; ET=60s | Embryo stage = 20ng  Cell line=20ng  Tissues=100ng | yes |
| 12 | Fw 6L_6  REV 12_11a | 6L and  11a | AT^1^=56°C; AT^2^=55°C; ET=60s | Embryo stage = 20ng  Cell line=20ng  Tissues=100ng | yes |
| Gapdh | Fw Gapdh  Rev Gapdh | N.A. | AT=55°C ET=30s ^c^ | Embryo stage = 20ng  Cell line=20ng  Tissues=20ng | [27] |
| Rn18s | Fw Rn18s  Rev Rn18s | N.A. | AT=55°C ET=30s ^c^ | Embryo stage = 20ng  Cell line=20ng  Tissues=20ng | [27] |

^a^AT^1^= annealing temperature 1 (°C); AT^2^= annealing temperature 2 (°C); ET=elongation time (s)

RT-PCR program: 4 min at 95°C; 18 Cycles of: [30 s at 95°C; 30s at AT^1^; ET at 72°C]; 22 Cycles of: [30 s at 95°C; 30s at AT^2^; ET at 72°C]

^b^When primer Rev 7_6 is used, in theory, two products (+ or - exon 6L) can be amplified. However, the elongation time was optimized for the smaller amplicon (-exon 6L).

^c^For amplification of household genes 25 cycles were sufficient.
